# Ketamine as a Novel Solution for Opioid-Refractory Pain in Radiation Necrosis

**DOI:** 10.7759/cureus.104578

**Published:** 2026-03-02

**Authors:** Courtney Campbell, Jamie Ellis-Wittenhagen, Tyler Murphy

**Affiliations:** 1 Palliative Medicine, Mayo Clinic, Phoenix, USA

**Keywords:** adolescent and young adult (aya), hospice and palliative care, ketamine infusion, palliative radiation therapy, radiation-induced necrosis, refractory cancer pain

## Abstract

We present the case of a 36-year-old female with recurrent metastatic melanoma, complicated by radiation necrosis of the left distal thigh musculature, who presented with intractable, debilitating pain approximately seven months following completion of local radiation therapy. Palliative Medicine was consulted for assistance with pain management. Prior to admission, the patient had failed to respond to escalating doses of oral oxycodone and transdermal fentanyl, given in conjunction with a multimodal regimen including acetaminophen, baclofen, celecoxib, duloxetine, and pregabalin. Just before presentation, the patient completed a prednisone taper for treatment of immune checkpoint inhibitor colitis, with suspicion that steroids were likely masking or partially treating pain before discontinuation. She was not a candidate for steroid re-initiation due to upcoming plans for tumor-infiltrating lymphocyte therapy. Despite escalating opioid dosing (up to oral morphine equivalent (OME) 598.75 mg), our patient continued to experience uncontrolled pain along with progressive sedation. We started her on ketamine 0.1 mg/kg/hr and titrated this over the next 24 hours to a final dose of 0.3 mg/kg/hr, which was then continued for 48 additional hours. Following completion of a three-day ketamine infusion, the patient's OME was reduced by 45% to 327.5 mg. In the months following hospital discharge, the patient was subsequently weaned off long-acting opioids and was able to return to her baseline functional status. No previous cases have been reported of the use of ketamine for the treatment of opioid-refractory pain secondary to radiation necrosis. In this case, we have demonstrated ketamine infusion as a successful treatment strategy for reducing our patient's opioid requirement, optimizing pain control, and helping to improve functional status and quality of life.

## Introduction

Radiation necrosis is a well-described complication of radiation therapy that develops due to permanent cell death in affected tissues and may result in significant localized pain. Corticosteroids comprise the primary treatment strategy [1]. As with other sources of cancer-associated pain, opioid therapy is a mainstay of providing symptomatic relief to patients. However, in some cases, patients may develop pain that is refractory to opioid therapy, prompting the need for alternative solutions to relieve potentially debilitating pain. Ketamine, a phencyclidine derivative, is an N-methyl-D-aspartate (NMDA) antagonist that can produce an analgesic effect at sub-anesthetic doses. This occurs by blocking NMDA-induced pain sensitization and increasing the sensitivity of opioid receptors [2]. This, in turn, can result in a reduction of opioid utilization. Increased investigation of the role the NMDA receptor plays in refractory pain has demonstrated that sustained input results in receptor activation, correlating with increased pain, expansion in the field of pain, and association with opioid resistance [3]. Ketamine has been studied in chronic pain, psychiatric disorders, and, more recently, in the treatment of sickle-cell pain. In general, findings suggest overall tolerability and reduction in opioid use [4]. Ketamine burst therapy refers to using ketamine as a continuous intravenous (IV) infusion for a short interval, followed by discontinuation, as an adjuvant approach to pain, and has been demonstrated in studies to successfully provide long-term analgesia in the setting of refractory pain [2,3]. Furthermore, research has shown that low-dose ketamine is safe and demonstrates the potential to reduce hospital admissions for intractable pain and, in turn, reduce healthcare costs [5]. Here, the experience using ketamine for the treatment of opioid-refractory pain secondary to radiation necrosis in a patient who was ineligible for corticosteroid therapy is presented. This article was previously presented as a meeting abstract at the 2025 Annual Assembly of Hospice and Palliative Care on March 12, 2025.

## Case presentation

The patient was a 36-year-old female with metastatic melanoma, initially diagnosed in 2016 with multiple subsequent recurrences. She had undergone numerous lines of therapy, including, most pertinent to this report, stereotactic body radiotherapy (SBRT) to the left posterior thigh, completed seven months prior to presentation. She subsequently developed focal radiation necrosis involving the distal biceps femoris and semitendinosus muscles. In the outpatient setting, the patient had failed to respond to escalating doses of opioid therapy consisting of a fentanyl transdermal patch 50 mcg/hour with as-needed (PRN) hydromorphone 4 mg every three hours, which she was utilizing around the clock, totaling an oral morphine equivalent (OME) of 260 mg, based on the equianalgesic conversions suggested by McPherson [6]. This was being given in conjunction with a multimodal regimen including acetaminophen 1000 mg twice daily, baclofen 20 mg three times a day, celecoxib 200 mg twice daily, duloxetine 30 mg daily, and pregabalin 100 mg twice daily.

She was instructed to increase her fentanyl patch to 75 mcg/hour. However, before this reached a therapeutic steady-state, she presented to the hospital with intractable pain in the left thigh. Of note, just before presentation, the patient had completed a prednisone taper for treatment of immune checkpoint inhibitor colitis, with suspicion that steroids had likely masked or partially treated the pain before discontinuation. She was not a candidate for steroid re-initiation due to upcoming plans for tumor-infiltrating lymphocyte (TIL) therapy. At the time of admission, the patient’s uncontrolled pain had resulted in significant impairment of her functional mobility, range of motion, gait stability, and ambulation. She reported that she had been nearly bedridden in the preceding days due to pain. Upon evaluation by physical therapy, she was noted to demonstrate limited mobility of the left lower extremity due to pain, only being able to ambulate approximately 10 feet with a three-point gait pattern. She was started on a demand-only hydromorphone patient-controlled analgesia (PCA) pump at 0.6 mg every 10 minutes, and she was continued on her fentanyl patch at the new dose of 75 mcg/hour. Palliative Medicine was consulted on hospital day two for assistance with pain management. By day four of hospitalization, her OME was 598.75 mg, with 398.75 mg from PRN medications. Despite this escalation, she continued to experience uncontrolled pain along with progressive sedation. She was subsequently started on a ketamine infusion, alongside an increase in her pregabalin dosing to 100 mg in the morning and 150 mg at night. We started an IV ketamine infusion at 0.1 mg/kg/hr on hospital day five and titrated this over the next 24 hours to a final dose of 0.3 mg/kg/hr, which was then continued for 48 additional hours. Her OME was 530 mg on hospital day five and decreased further to 327.5 mg by the time the ketamine infusion was completed on day eight, with her PRN opioid utilization decreasing from 308.75 mg to 127.5 mg over this same timeframe. This reflected a reduction of her total OME of 45.3% and a reduction in PRN utilization by 58.7%.

At the time of hospital discharge on day nine, the patient was able to ambulate with contact guard assist of one with a front-wheeled walker for approximately 50 feet. She was discharged on a fentanyl patch of 100 mcg/hr with hydromorphone 6-8 mg every three hours PRN for breakthrough pain. The patient was followed up with as an outpatient for three months following hospital discharge prior to her moving back to her home state. Within the first two months after discharge, she weaned off long-acting opioids completely and was able to return to her baseline functional status.

## Discussion

Radiation-induced necrosis often results in severe neuropathic pain that is notoriously refractory to standard analgesic regimens, including opioids, anticonvulsants, and steroids. Our patient's persistent pain, despite escalating opioids and adjuvants, highlights the need for alternative strategies in these complex cases. No previous cases have been reported of the use of ketamine for the treatment of opioid-refractory pain secondary to radiation necrosis [7].

Ketamine, a non-competitive NMDA receptor antagonist, offers a compelling option in such contexts. Its use in refractory cancer and neuropathic pain is supported by both case reports and prospective studies; one observational series showed significant pain relief and reduced opioid requirements in 74% of patients treated with ketamine infusions, while individual case reports document effective use in radiation-induced brachial plexopathy and advanced cancer-related neuropathic pain [8].

In this case, we have demonstrated that ketamine infusion resulted in a rapid and durable improvement in pain control, alongside measurable opioid sparing. The tolerability profile in our case (i.e., minimal psychotomimetic effects, stable hemodynamics) aligns with broader evidence that sub-anesthetic dosing (<0.5 mg/kg) is effective and safe when titrated appropriately and monitored. While dissociative side effects are possible, they were not observed here, consistent with other reported series [9].

## Conclusions

Ketamine infusion is an effective treatment plan for patients suffering from pain related to radiation-induced necrosis, especially in cases refractory to standard lines of treatment. Ketamine infusion is a relatively well-tolerated treatment plan, especially at sub-anesthetic dosing. More studies are required to investigate the pathways of pain in radiation necrosis and explore the role of ketamine as an adjuvant in pain management in these cases.

## References

[1] Delanian S, Lefaix JL (2007). Current management for late normal tissue injury: radiation-induced fibrosis and necrosis. Semin Radiat Oncol.

[2] Nath TS (2022). Effectiveness of low-dose ketamine infusion in opioid refractory cancer pain: a case report. Cureus.

[3] Jackson K, Ashby M, Martin P, Pisasale M, Brumley D, Hayes B (200122). “Burst” ketamine for refractory cancer pain: an open-label audit of 39 patients. J Pain Symptom Manage.

[4] Onyebuchi CO, Chumpitazi CE, Placencia JL, Jackson AN, Jones JL, Torres L, Tubman VN (2024). Ketamine for pain in sickle cell disease reduces opioid usage. J Pain Symptom Manage.

[5] Loveday BA, Sindt J (2015). Ketamine protocol for palliative care in cancer patients with refractory pain. J Adv Pract Oncol.

[6] McPherson ML (2018). Demystifying Opioid Conversion Calculations: A Guide for Effective Dosing.

[7] Kurosaki F, Takigami A, Takeuchi M, Shimizu A, Tamba K, Bando M, Maemondo M (2024). Successful pain control with add-on methadone for refractory neuropathic pain due to radiation necrosis in pontine metastatic lesion: a case report. BMC Palliat Care.

[8] Chai T, Truong N, Roldan CJ (2020). Refractory painful brachial plexopathy due to radiation therapy relieved with serial intravenous ketamine infusions: a case report. PM&R.

[9] Tarumi Y, Watanabe S, Bruera E (2000). High-dose ketamine in the management of cancer-related neuropathic pain. J Pain Symptom Manafe.

